# Basal Ganglia Activity Mirrors a Benefit of Action and Reward on Long-Lasting Event Memory

**DOI:** 10.1093/cercor/bhv216

**Published:** 2015-09-28

**Authors:** Raphael Koster, Marc Guitart-Masip, Raymond J. Dolan, Emrah Düzel

**Affiliations:** 1 Institute of Cognitive Neuroscience, University College London, London WC1N 3AR, UK; 2 Wellcome Trust Centre for Neuroimaging, Institute of Neurology, University College London, London WC1N 3BG, UK; 3 Aging Research Centre, Karolinska Institute, SE-11330 Stockholm, Sweden; 4 Otto von Guericke University Magdeburg, Institute of Cognitive Neurology and Dementia Research, D-39120 Magdeburg, Germany; 5 German Center for Neurodegenerative Diseases, D-39120 Magdeburg, Germany; 6 Max Planck Centre for Computational Psychiatry and Ageing, University College London, London, UK

**Keywords:** decision-making, fMRI, hippocampus, Pavlovian

## Abstract

The expectation of reward is known to enhance a consolidation of long-term memory for events. We tested whether this effect is driven by positive valence or action requirements tied to expected reward. Using a functional magnetic resonance imaging (fMRI) paradigm in young adults, novel images predicted gain or loss outcomes, which in turn were either obtained or avoided by action or inaction. After 24 h, memory for these images reflected a benefit of action as well as a congruence of action requirements and valence, namely, action for reward and inaction for avoidance. fMRI responses in the hippocampus, a region known to be critical for long-term memory function, reflected the anticipation of inaction. In contrast, activity in the putamen mirrored the congruence of action requirement and valence, whereas other basal ganglia regions mirrored overall action benefits on long-lasting memory. The findings indicate a novel type of functional division between the hippocampus and the basal ganglia in the motivational regulation of long-term memory consolidation, which favors remembering events that are worth acting for.

## Introduction

Episodes that are experienced at any given moment can usually be remembered a few minutes later, but only a fraction of episodes are consolidated as long-lasting memories. Why some episodes are consolidated and others forgotten is still not fully understood. Converging evidence points toward the importance of motivational valence (Shohamy and Adcock 2010; Lisman et al. 2011), because events that predict rewards are more likely to be remembered after long delay intervals (24 h and longer) than those associated with neutral outcomes (Wittmann et al. 2005, 2011; Adcock et al. 2006). However, recent studies indicate that valence representations are also closely coupled to action requirements (Crockett et al. 2009; Guitart-Masip et al. 2011, 2012, 2014; Levita et al. 2012), suggesting that the explanatory power of valence in isolation could be limited. Therefore, instead of valence, memory performance may depend on whether rewards are obtained through action or inaction.

Motivational influences on episodic long-term memory are likely to be mediated by functional anatomical loops between the hippocampus and the dopaminergic neurons (Morris 2006; Hansen and Manahan-Vaughan 2014) in the substantia nigra/ventral tegmental area (SN/VTA; Lisman and Grace 2005; Lisman et al. 2011). Pharmacological studies in rats (O'Carroll et al. 2006) and aging humans (Chowdhury et al. 2012) have shown that dopamine can improve hippocampal-dependent memory consolidation. Likewise, reward anticipation (Wittmann et al. 2005,  2011; Adcock et al. 2006; Callan and Schweighofer 2008; Wolosin et al. 2012) boosts memory consolidation via coactivation (Wittmann et al. 2005) and increased functional connectivity (Adcock et al. 2006) of hippocampus and SN/VTA. In human studies of reward anticipation, the need to perform an impending action has been shown to dominate brain responses in the striatum and the SN/VTA and has a greater impact here than the anticipation of expected value (Guitart-Masip et al. 2011). Together with previous observations (Tricomi et al. 2004; Zink et al. 2004), these findings show that motivational brain activity is strongly associated with the anticipated action needed to obtain a reward. Evidence for this intrinsic coupling of anticipated action and reward (Dayan et al. 2006) in dopaminergic circuitry (Guitart-Masip et al. 2011) is also seen following pharmacological manipulations (Guitart-Masip et al. 2012).

**Figure 1.**
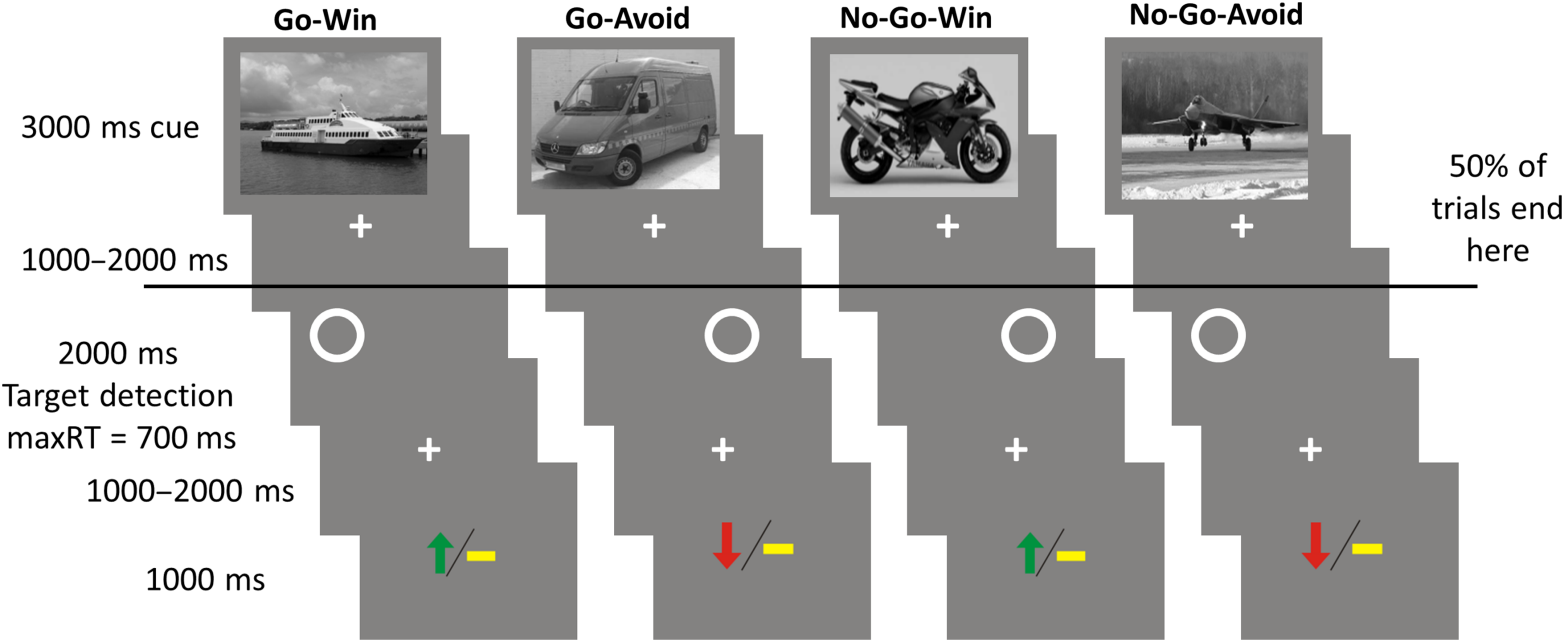
Experimental paradigm performed in the scanner. Subjects were presented 80 trials in each of 4 consecutive blocks. In each trial, a trial-unique image from 1 of 4 categories (randomized across participants) informed subjects about the 2 factors: Whether to respond by indicating the position of a circle with an either left or right button press or to omit a response entirely (Go or No-Go); whether they could win a reward or avoid a loss by performing a correct action or inaction (Win or Avoid). On Win trials, subjects could win £1 if they performed correctly, or gain £0 if they performed incorrectly. On Avoid trials, subjects could gain £0 instead of losing £1 by performing correctly. The outcomes after a correct response were 70% probabilistic. To disassociate anticipation from actual performance, half of the trials ended after the cue image and were directly followed by a new cue image. Subjects were trained extensively with a different set of images that were not trial unique before entering the scanner.

These insights regarding the relationship between action and valence can provide new perspectives on the motivational regulation of memory consolidation. For example, asymmetry in memory persistence based on the congruence of action and reward could be adaptive in an evolutionary sense because an action that led to reward may be the most valuable information to retain, especially compared with rewards that are gained passively. Additionally, value representations themselves may be affected by an association with action (Schonberg et al. 2014), which also could enhance the memory benefit further. We hypothesized that the need to perform an impending action would modulate memory consolidation beyond any effects attributable to reward expectation per se. To address this, we adapted a design (Guitart-Masip et al. 2011, 2012) that orthogonalizes the anticipation of action and valence to the context of a memory encoding experiment while we acquired simultaneous fMRI data (Fig. 1). This allowed us to investigate whether action, reward valence, or an interaction of the two affected memory persistence. The design also allowed to contrast reward for action and identical reward for inaction. We were particularly interested in determining whether the ability to remember new information under the anticipatory influence of reward and action requirement would be determined by the hippocampus, by the basal ganglia and the SN/VTA (i.e., Chowdhury et al. 2012), or both. A behavioral follow-up study was conducted with a 30-min delay between encoding and retrieval in order to investigate the time course of the memory modulation.

## Methods

### Participants

Thirty-three healthy right-handed participants who provided informed consent took part in the study. All had normal or corrected-to-normal vision and no history of psychological or neurological illness. One participant was excluded for poor overall memory (overall corrected hit rate below 0) and 3 were excluded for signal dropout in the midbrain (detection of loss of inferior SN/VTA regions was guided by a priori anatomical masks), leaving 29 participants (12 male, 18–31 years, 26.3 years mean age). A behavioral follow-up study with the 30-min delay between sessions contained 30 subjects (13 males, 18–34 years, 22 years mean age). The study was approved by the Institute of Neurology (University College London) Research Ethics Committee. Participants received reimbursement for their participation.

### Stimuli

Stimuli consisted of 480 grayscale images of cars, boats, motorbikes, and airplanes (320 used in the task and 160 used as lures for the memory task). The stimuli were presented in randomized order on a gray background using Cogent 2000 (www.vislab.ucl.ac.uk/cogent.php) running in MATLAB.

### Experimental Protocol

Two experiments were conducted in 2 independent samples: 1 fMRI experiment and 1 behavioral follow-up. Each study consisted of 2 sessions. Session 1 (Fig. 1) consisted of 4 experimental runs during which functional MRI data were acquired (for the follow-up group, the experiment was conducted with no scanning, simply in front of a computer). Each run consisted of 80 trials. Each trial consisted of the 3 following events: Presentation of a trial-unique image (of 1 of 4 image categories: cars, boats, motorbikes, and airplanes) for 3000 ms; the display of a circle for 2000 ms on either the left or right side of the screen; the display of the outcome for 1000 ms represented by either a green arrow pointing up (win), a yellow horizontal bar (neutral), or a red arrow pointing down (loss). Each of the 3 events was followed by a fixation cross for a variable interval of 1000–2000 ms. Half of the trials were aborted after the fixation cross following the display of the image. This allows to decorrelate the encoding event (and anticipation of action and reward from) from the actual performance and outcome (however not performance and outcome from each other).

The image category instructed the participant whether on this trial it would be required to indicate the position of the circle with a keypress (Go) or omit the response entirely (No-Go). The Go response had to be entered in under 700 ms in order to be registered. The images also informed the participant about the potential valence of the outcome: win/neutral (Win condition) or neutral/loss (Avoid condition). In each trial, £1could either be won or lost. The outcome was probabilistic to 70% for correct responses (e.g., 30% of correct Win trials produced a neutral outcome) and deterministic for incorrect responses. The mapping of the image categories to the experimental conditions was counterbalanced across participants. Because wins and avoided losses were of equal magnitude in absolute terms for the Go and No-Go conditions, this design holds expected value constant within each action condition enabling us to separately probe the effect of action and outcome valence. The 4 conditions can be considered of high salience because they all signal a potentially affective outcome (Bromberg-Martin et al. 2010; Guitart-Masip et al. 2014). Note that due to the outcomes being probabilistic subjects faced the whole range of outcomes even at perfect performance.

Each of the 4 experimental runs contained 20 trials per condition (Go Win, Go Avoid, No-Go Win, and No-Go Avoid), half of which were aborted after the display of the image and did not require the Go/No-Go response.

Subjects were instructed that their performance across all stages of the experiment would affect their payment, resulting in a total outcome of up to £45. To ensure that subjects learned the meaning of the image categories, subjects completed 3 runs of training. The first run consisted of 5 trials in which subjects were asked to indicate the position of the circle with a button press. The second run was 1 block in which no trial was aborted after image display, and the outcome was accompanied by text providing feedback whether the response was correct. To ensure subjects were familiar with the manipulation by which half of the trials were aborted, another run of training consisted of a shortened version of an actual experimental run (∼30 min of total training time). During the training the images were not trial unique but a repeating subset, that was not used in the actual experimental runs. See Guitart-Masip et al. (2011) for a similar experimental paradigm in which fractal images were used instead of image categories to indicate the condition of the trial. Note that, in this paradigm, the instructions are semantically embedded in the memoranda (images of vehicles). Reactions can only be planned post perception and semantic classification of the stimulus. This ensures a lower bound of equal encoding across all conditions.

Session 2 took place the following day for the fMRI group and after a 30-min break for the behavioral follow-up experiment group. Subjects were instructed in advance that there would be a second session but not what type of task the second session would include. Subjects were presented with all 80 images of each image category they saw in the experimental runs of Session 1 plus 40 images of each category they had not seen before (this included cue images of trials in which the response and outcome parts of the trial were skipped). For each image, subjects were asked to indicate whether they had seen the image in Session 1 or not. After that, subjects were asked to rate their confidence in their answer as either “very sure,” “sure,” or “uncertain.” If subjects responded they saw the image in Session 1, the additional confidence option “remember” was available. Subjects were asked to use this option if they had a vivid recollection of the full context in which they saw the image. Furthermore, if subjects responded they saw the image in Session 1, they were also asked whether or not the image was directly followed by a circle. Images were presented in randomized order. There was no reaction time limit for any of the questions in Session 2 (session duration ∼50 min).

### MRI Data Acquisition

The study was conducted at the Wellcome Trust Center for Neuroimaging at University College London using a 3-T Siemens Trio scanner equipped with a 32-channel Siemens head coil.

### Anatomical fMRI Data Acquisition

For subjects whose anatomical images were not previously available, multiparameter maps were acquired. A multiparameter map protocol (Weiskopf et al. 2013) using a 3D multi-echo fast low-angle shot (FLASH) sequence with 1-mm isotropic resolution was used to acquire magnetic transfer-weighted images [echo time (TE) = 2.2–14.70 ms; repetition time (TR) = 23.7 ms; flip angle (FA) = 6°)], *T*_1_-weighted images (TE = 2.2–14.7 ms; TR = 18.7 ms; FA = 20°), and proton-density-weighted images (TE = 2.2–19.7 ms; TR = 23.7 ms; FA = 6°). B1 mapping (TE = 37.06 and 55.59 ms; TR = 500 ms; FA = 230:−10:130°; 4 mm^3^ isotropic resolution) was acquired to correct the *T*_1_ maps for inhomogeneities in the transmit radiofrequency field (Lutti et al. 2010). A double-echo FLASH sequence (TE1 = 10 ms; TE2 = 12.46 ms; 3 × 3 × 2 mm resolution; 1 mm gap) was used to measure local field inhomogeneities and correct for the image distortions in the B1 mapping data.

### Functional MRI Data Acquisition

Functional scans used a gradient-echo sequence optimized for hippocampus coverage: TR (per volume) = 2.975 s; TE = 37 ms; 2.3 mm isotropic image resolution; interleaved acquisition order; matrix size = 96 × 96; field of view = 192 × 222 mm. A total of 35 axial slices (−45° tilt) were sampled for partial brain coverage including the hippocampus and the striatum.

A field map was recorded using a double-echo FLASH sequence (matrix size = 64 × 64; 64 slices; spatial resolution: 3 × 3 × 2 mm; 1 mm gap; TE1 = 10 ms; TE2 = 12.46 ms; TR = 1020 ms) for distortion correction of the acquired functional images. Field maps were estimated from the phase difference between the images acquired at the short and long TE with the FieldMap toolbox for SPM8.

Participants' pulse and breathing were measured synchronized with MRI scanner slice pulses using the Spike2 data acquisition system (Cambridge Electronic Design Limited, Cambridge, UK). The cardiac pulse signal was measured using an pulse oximeter (Model 8600 F0, Nonin Medical, Inc., Plymouth, MN, USA) attached to the participants' left index finger. Thoracic movement was recorded using a pneumatic belt positioned around the abdomen.

### Analysis of Behavioral Data

The data were analyzed using Matlab R2012b and SPSS 19. Response speed in Go trials was analyzed by testing the speed of correct Go responses between the 2 Go conditions (Go-Win vs. Go-Avoid) with a paired *t*-test. The number of correct Go/No-Go responses per condition was analyzed with a 2 × 2 repeated-measures ANOVA with action (Go/No-Go) and valence (Win/Avoid) as factors. As there were not enough “remember” responses for an fMRI analysis, a memory score was calculated that weighted correct and incorrect responses according to their confidence (e.g., a correct recognition response with a “remember” confidence rating added 4 points to the total while an incorrect recognition response with a “sure” confidence rating subtracted 2 points from the total). The memory score was analyzed with a 2 × 2 repeated-measures ANOVA with action (Go/No-Go) and valence (Win/Avoid) as factors. For the group comparison, a between-subject factor was added. Post hoc *t*-tests were performed to further analyze these effects.

### fMRI Data Preprocessing

Imaging data were analyzed with Statistical Parametric Mapping (SPM8; Wellcome Trust Centre for Neuroimaging, London, UK; http://www.fil.ion.ucl.ac.uk/spm). Images were bias-corrected for intensity inhomogeneities, realigned with the first volume (after discarding the first 6 dummy volumes) and unwarped, co-registered with the structural image, normalized to a standard echo-planar imaging template based on the Montreal Neurological Institute reference brain, resampled to 2 × 2 × 2 mm^3^ voxels, and spatially smoothed (4-mm full-width at half-maximum). The magnetization transfer images were averaged across subjects after normalization in order to identify the SN/VTA and create a group-specific template (Duzel et al. 2009).

### fMRI Data Analysis

The fMRI data were analyzed in a 2 × 2 [Action (Go/No-Go) × Valence (Win/Avoid)] design to mirror the behavioral analysis. For each participant, a statistical model was computed by applying a canonical hemodynamic response function combined with time and dispersion derivatives. The time series indicated the temporal position of: display of image for each of the 4 conditions (Go Win, Go Avoid, No-Go Win, and No-Go Avoid), the onset of the circles (for each Go and No-Go trials in which subjects correctly responded), display of outcomes for the 3 possible outcomes (win, neutral, and loss), and display of image for trials with an incorrect response. Note that this includes the trials that were aborted after the cue image. To mirror the behavioral analysis of the data, each of the 4 conditions indicating the cue onset was parametrically modulated by memory strength (ranging from “not seen before–very sure” to “seen before–remembered”). To remove physiological noise, a model was constructed to account for artifacts related to cardiac and respiratory phase and changes in respiratory volume using an in-house developed Matlab toolbox (Hutton et al. 2011). A total of 14 regressors for physiological noise reduction and 6 motion correction regressors estimated from the realignment procedure were entered as covariates of no interest (physiological data were available for all but 2 participants, for whom just 6 motion correction regressors were entered). Contrast images were entered into two 2 × 2 ANOVAs (Action by Valence) for the second-level random-effects analysis, one containing the contrasts of the 4 conditions coding the image onset (cue activity) and the other containing the 4 parametric modulators of memory strength for each of those conditions (memory parameter). The main effect of memory was assessed by weighting all 4 conditions containing the memory parameter over baseline. The main effects of action (and its inverse for inaction) and valence were assessed for both second-level models. All these effects were assessed at the whole-brain FWE cluster-corrected level after voxel-wise thresholding *P* < 0.001. The memory contrast (all memory parameters against baseline) was masked with bilateral anatomical masks of the hippocampus. Furthermore, we investigated the interaction of action and valence in the memory parameter using a small-volume correction for a bilateral anatomical mask including the hippocampus and the basal ganglia (after thresholding at *P* < 0.001 uncorrected) due to their role in memory and motivation. For all clusters of interest, the average activation over all voxels in the respective functional regions of interest (ROIs) was extracted using the MarsBaR toolbox (Brett et al. 2002) and analyzed using a 2 × 2 ANOVA (Action by Valence). Post hoc *t*-tests were used to investigate the precise differences across conditions that created the observed effects in the ANOVAs.

For all parts of the fMRI analysis, the WFU PickAtlas toolbox for SPM was used to identify the anatomical location of peak voxels (Lancaster et al. 2000; Maldjian et al. 2003). In the case of the SN/VTA, the anatomical mask was created based on the average magnetization transfer image of all subjects. The average activation over all voxels in the individual ROIs was extracted using the MarsBaR toolbox (Brett et al. 2002).

## Results

**Figure 2.**
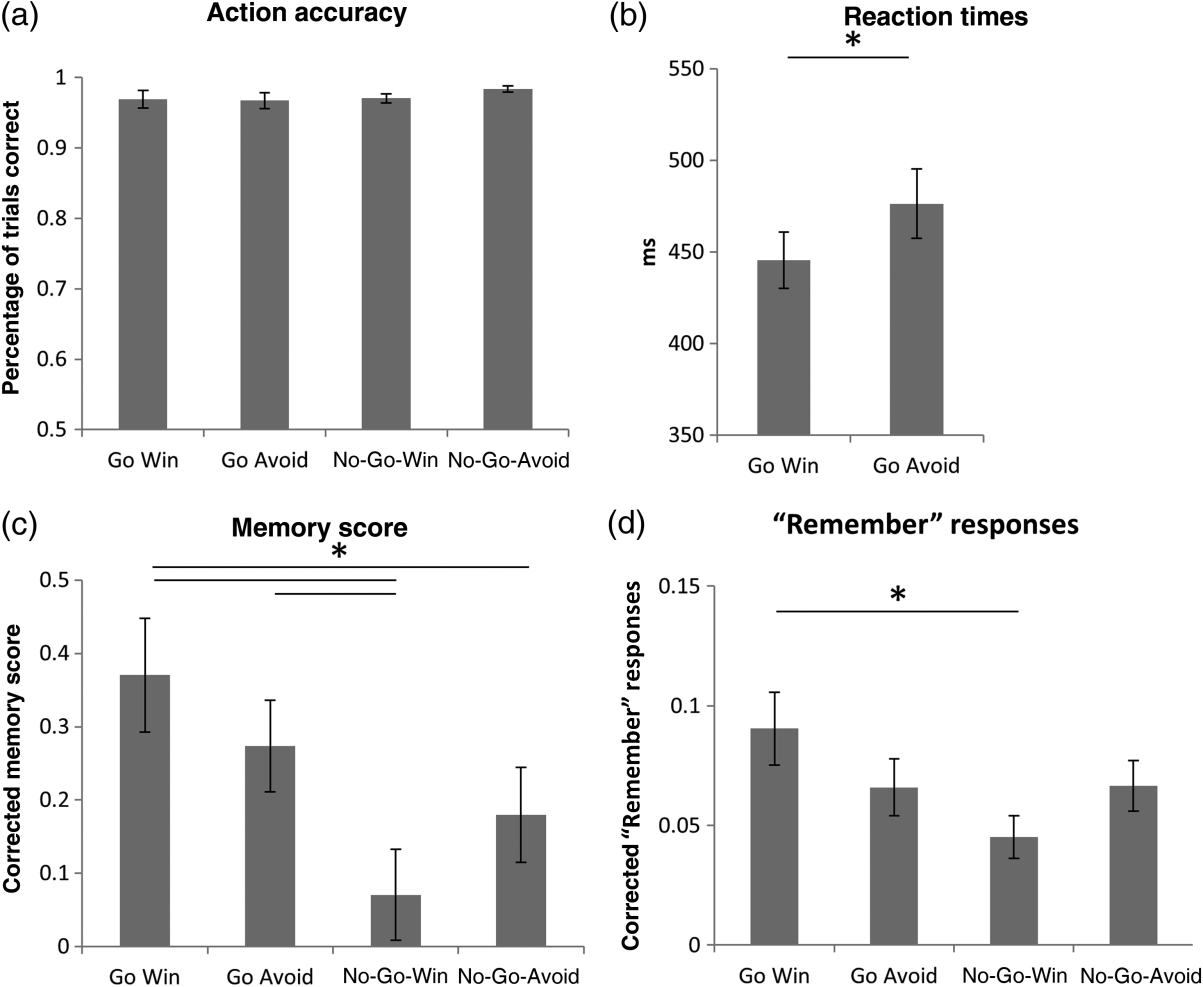
(*a*) Action accuracy (correctly performing Go or No-Go) did not differ across conditions. (*b*) Subjects' responses were faster for Go Win than for Go Avoid trials. (*c*) The memory score (correct vs. incorrect recognition weighted by confidence) differs across conditions, showing both an enhancement of action and an interaction of action and valence. (*d*) The “Remember” responses follow the same pattern as the memory score. Post hoc tests were performed by means of paired *t*-test. Significant differences (*P* < 0.05) are marked by an asterisk. Error bars represent standard error of the mean.

During the scanning session, subjects' responses (Go or No-Go) after the cue were 97.2% correct and this did not differ across conditions (Fig. 2*a*, main effect of action: *F*_1,28_ = 1.05, *P* > 0.3; main effect of valence: *F*_1,28_ = 1.79, *P* > 0.1; interaction: *F*_1,28_ = 1.25, *P* > 0.2). Correct Go responses were significantly [*t*_(28)_ = 3.83, *P* = 0.001] faster in Go Win (445.43 ± 82.23 ms) than in Go Avoid trials (476.32 ± 102.07 ms), indicating that participants anticipated the valence associated with the different conditions along with the associated action requirements (Fig. 2*b*). As in previous versions of the task (Guitart-Masip et al. 2011, 2012), the number of responses that were correct yet below the instructed 700 ms threshold was higher in the Go Win compared with the Go Avoid condition [*t*_(28)_ = 2.92, *P* = 0.007]. The reaction time facilitation by reward valence demonstrates that subjects processed valence, even though valence information was not instrumentally essential in expressing the correct action requirements.

### The Behavioral Effect of Action and Valence on Memory After 24 h and 30 min

Twenty-four-hour memory accuracy across conditions was assessed by calculating a memory score that weighted answers according to their confidence judgment and added or subtracted from the total depending on whether the answer was correct. A positive score means that correct answers were given more confidently and/or frequently than wrong answers. As shown in Figure 2*c*, memory score showed both a main effect of action (*F*_1,28_ = 12.54, *P* = 0.001) and an interaction (*F*_1,28_ = 4.7, *P* = 0.039) but no effect of valence (*F*_1,21_ = 0.1, *P* > 0.9). These effects were driven by the Go versus No-Go difference between the Win conditions [*t*_(28)_ = 3.65, *P* = 0.001] and the lack of a Go versus No-Go difference in the Avoid losing conditions [*t*_(28)_ = 1.51, *P* > 0.1]. The Go Win condition also showed better memory compared with the No-Go Avoid condition [*t*_(28)_ = 2.32, *P* = 0.028], and the No-Go Win condition showed significantly lower memory performance than the Go Avoid condition [*t*_(28)_ = 3.1, *P* = 0.005]. This score also contained “Remember” responses, which showed a highly similar pattern [Fig. 2*d*; main effect of action (*F*_1,28_ = 4.89, *P* = 0.035) and an interaction (*F*_1,28_ = 4.6, *P* = 0.041); Go Win vs. No-Go Win (*t*_(28)_ = 2.79, *P* = 0.009); see Supplementary Materials for recognition memory]. Note that we found no connection between reaction time to the action cue and subsequent memory.

The behavioral experiment was repeated without fMRI scanning but now with a 30-min delay (instead of 24 h) in order to determine whether the effect of action on memory was already present even after a short delay. This would be compatible with a strong effect of action on encoding. When the experiment was repeated with a 30-min delay, the main effect of action did not replicate (though showed a trend-level effect, *F*_1,29_ = 2.74, *P* = 0.11), and the interaction of action and valence did replicate (*F*_1,29_ = 13.11, *P* = 0.001). Testing the effect of delay length on the effect of action or the interaction of action and valence yielded no significant results. Thus, although we do not see statistically significant group differences, these data indicate that a strong behavioral main effect of action observed after 24 h is not explained as a residue of a stronger encoding modulation by action visible after 30 min.

### fMRI Analyses

To examine how the experimental conditions affect memory encoding at a neural level, we analyzed the blood oxygen level-dependent activity evoked by image onset (hereafter “cue activity”). The data were analyzed in a 2 × 2 ANOVA containing the factor of action (Go/No-Go) and valence (Win/Avoid). The same ANOVA was constructed with the parametric modulation of each of the 4 conditions, containing a 7-point subsequent memory rating ranging from “sure not seen before” to “remembered seen before.” This activation parametrically reflected activity correlated with memory fate after 24 h (hereafter “memory parameter”). Note that these 2 measures are independent of each other and can be interpreted separately: While the cue activity describes the overall activity in a condition (like an intercept), the memory parameter describes how much the activity increases as a function of subsequent memory (like a slope).

The factorial design and the orthogonal nature of the contrasts allowed us to employ a functional ROI approach that extracts the beta estimates of all conditions from regions that have been independently selected. The fMRI analyses were conducted on statistical maps (thresholded at *P* < 0.001 uncorrected) that were corrected for multiple comparisons on the whole-brain level or for a bilateral anatomical ROI including the basal ganglia and the hippocampus together.

### Neural Effects of Action, Inaction, and Valence

**Figure 3.**
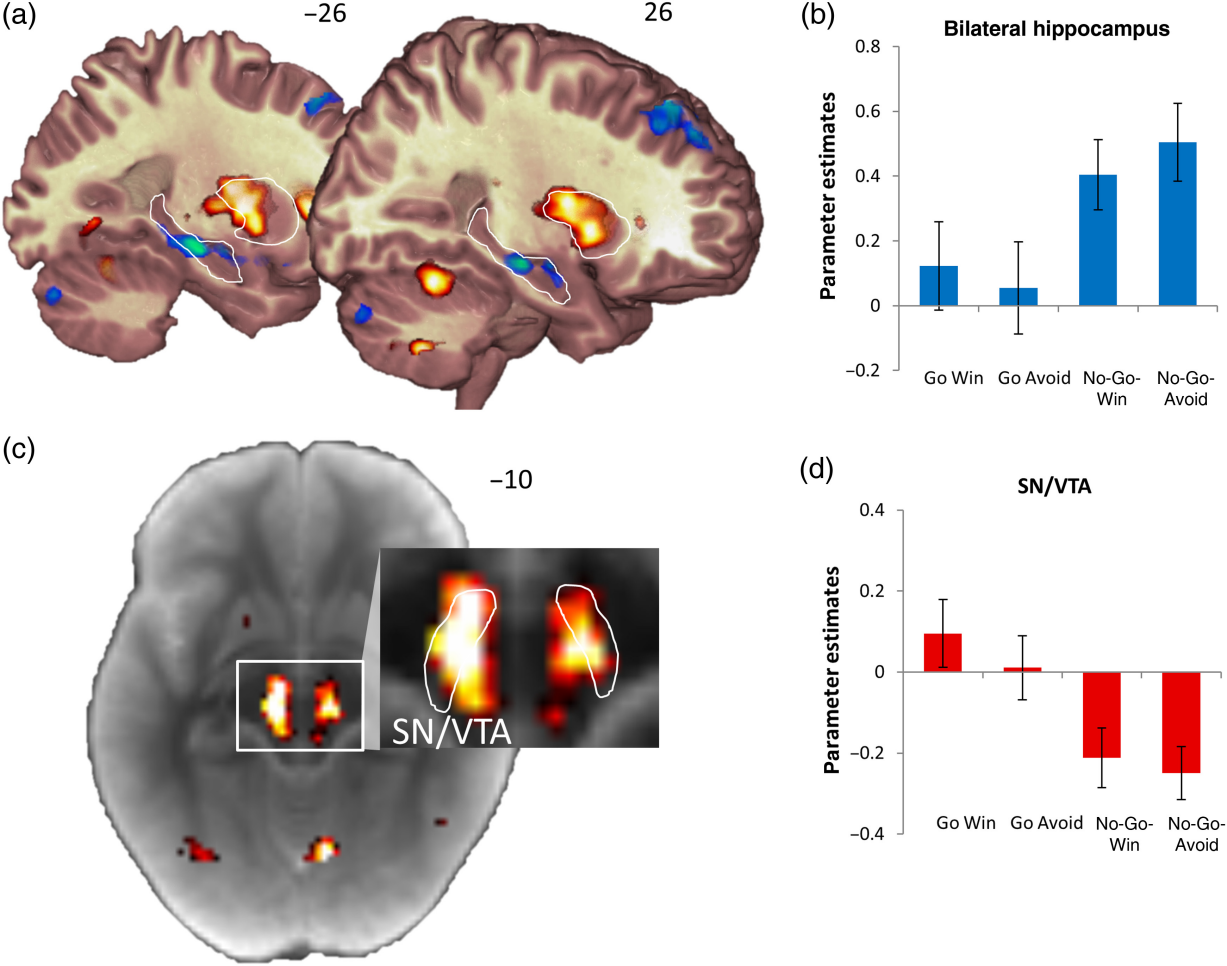
(*a*) The main effects of action (red) and inaction (blue) on cue activity. The bilateral hippocampus and putamen are outlined for display purposes. (*b*) The bilateral hippocampus shows increased activation during the anticipation of inaction. (*c*) To localize the activity in the midbrain, the statistical map of the main effect of action was displayed on the average magnetization transfer weighted image of the subjects. The SN/VTA is within the outlined hyperintense stripe (Duzel et al. 2009). The contrast of the magnified display is optimized to differentiate midbrain structures. (*d*) The activation of the midbrain cluster shows a main effect of action. All activations are thresholded at *P* < 0.001 uncorrected. Error bars represent standard error of the mean.

Examining the cue activity in the contrast of Go over No-Go (action) and its inverse (inaction) revealed large sets of significant clusters (see Supplementary Materials for full list). Most dominantly, the effect of action revealed large whole-brain-corrected clusters spanning the midbrain, the thalamus, and the entire striatum (peaking at −14 −18 16, *z* = 7.34, *k* = 4718, Fig. 3). There were no whole-brain-corrected effects of valence anticipation. This activation pattern replicated previous findings (Guitart-Masip et al. 2011, 2012). However, a positive effect of valence was present in the action cluster in the SN/VTA (Fig. 3*c*,*d*, *F*_1,28_ = 4.81, *P* = 0.037). This result shows that the statistical power of the current study can uncover action-independent reward representations, which are dominated be action representations (Guitart-Masip et al. 2011).

The inaction contrast revealed large bilateral hippocampal clusters (whole-brain FWE-corrected at the cluster level at *P* < 0.05, left peaking at −26 −22 −12, *z* = 4.91, *k* = 239 and right peaking at 26 −20 −12, *z* = 4.77, *k* = 101, Fig. 3*a*,*b*) and also 2 large clusters including the amygdala and the lateral temporal cortex (left peaking at −60 −14 −6, *z* = 6.07, *k* = 2322 and right peaking at 38 −16 −22, *z* = 6.33, *k* = 2890).

### Modulation of Subsequent Memory Effects by Action and Valence

First, we identified brain regions which showed a main effect of subsequent memory (memory parameter averaged across all conditions). This analysis resulted in significant clusters in medial temporal and frontal lobes (see Supplementary Materials for full list). Memory parameters from each of the 4 experimental conditions were extracted from these clusters to investigate the effects of action and valence on memory. However, we found that within these clusters, memory parameters did not differ across conditions (all main effects and interactions not significant). Thus, in regions selected for a subsequent memory effect, the activity difference between subsequently recognized/remembered items and those items that were forgotten did not differ across action and valence conditions. As the hippocampus was of specific interest here due to its central role in memory (Vargha-Khadem et al. 1997; Eichenbaum et al. 2007; Ranganath 2010; Squire and Wixted 2011), we anatomically restricted the activation map showing a significant effect of memory parameter across conditions to bilateral hippocampi (note that this cluster remained significant whole-brain FWE-corrected). While this hippocampal cluster showed a positive memory parameter in every condition except No-Go Win, we again found no significant differences across action/valence conditions for the memory parameter within the hippocampus (see Supplementary Materials for details). While the hippocampus reflected subsequent memory, its overall activity levels showed increased activity in the No-Go conditions (see above for effect of inaction), suggesting that the hippocampal activity may not alone account for the observed behavioral memory performance differences across conditions.

**Figure 4.**
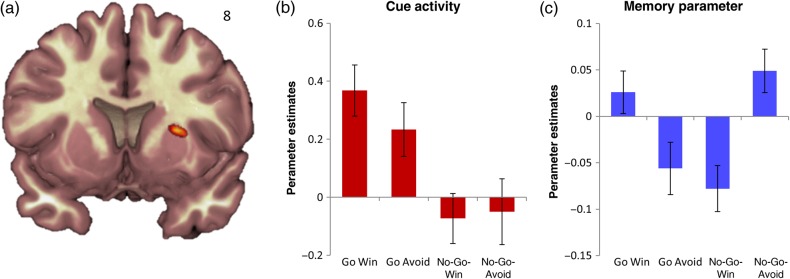
(*a*) The interaction of action and valence of the memory parameter is displayed at *P* < 0.001 uncorrected. The betas of the putamen cluster for cue activity (*b*) and the memory parameter (*c*) were extracted. Error bars represent standard error of the mean. The interaction effect shows a Pavlovian congruence effect favoring action for reward and inaction for avoidance of loss.

Therefore, we conducted a more specific analysis that directly targeted the interaction of the memory parameter with action and valence. On the basis of previous work on the relationship between memory and motivation (Haber and Knutson 2010; Guitart-Masip et al. 2011; Chowdhury et al. 2012), this analysis was restricted to the hippocampus and the basal ganglia. Investigating the interaction effect on the memory parameter in the hippocampus and basal ganglia revealed a significant cluster in the right putamen (Fig. 4, FWE small-volume correction for a priori anatomical ROI consisting of the hippocampus and basal ganglia peaking at 26 8 8, *P* = 0.037, *z* = 4.27, *k* = 32). Of course, the finding of a neural interaction effect does not mean that this signal is necessary to produce the behavioral results. For the memory parameter, both congruent conditions (Go Win and No-Go Avoid) were significantly increased compared with the incongruent conditions [Go Win vs. Go Avoid: *t*_(28)_ = 2.12, *P* = 0.043; Go Win vs. No-Go Win: *t*_(28)_ = 3.05, *P* = 0.005; No-Go Avoid vs. Go Avoid: *t*_(28)_ = 2.58, *P* = 0.016; No-Go Avoid vs. No-Go Win: *t*_(28)_ = 3.83, *P* < 0.001]. The cue activity in this cluster revealed a main effect of action (*F*_1,28_ = 13.28, *P* < 0.001, Fig. 4*b*) and a trend of an interaction (*F*_1,28_ = 4.1, *P* = 0.053).

## Discussion

We show that images that predict a necessity to act were more likely to be recognized and remembered after a long retention interval (24 h) than those that predicted a necessity to omit action. This action benefit was particularly strong for images associated with reward but there also was a main effect of action across both the Win and Avoid conditions (Fig. 2*c*). Considering images associated with vivid recollection showed a highly similar pattern of an enhancement by action and the interaction of action and valence (remember responses to Go Win vs. No-Go Win, Fig. 2*d*).

It is well established that the ability to recognize events with high confidence and to experience vivid recollection is critically dependent on the hippocampus (Vargha-Khadem et al. 1997; Eichenbaum et al. 2007; Ranganath 2010; Squire and Wixted 2011). However, the observed dominant hippocampal activation for anticipated omission of action constitutes a striking dissociation between hippocampal activity at the time of encoding and behavioral memory. This dissociation and the fact that covariation of subsequent memory with hippocampal activation was constant across conditions make it unlikely that either the valence-independent memory benefit for anticipated action or the improved recollection for Go Win was driven by a hippocampal enhancement of encoding.

Taken together, the valence-independent enhancement of long-term memory in the Go conditions cannot be fully explained by hippocampal encoding processes per se. Rather, the long-term memory performance is compatible with an enhancement of memory consolidation independent of hippocampal activation during encoding (Chowdhury et al. 2012). Indeed, a follow-up experiment using the same task design, but a short retention interval of 30 min, enables us to rule out the possibility that a benefit we observed after 24 h is a residue of a stronger immediate effect, which would likely reflect an encoding benefit.

Given the behavioral memory enhancement by action and the involvement of dopamine in long-term memory consolidation (Chowdhury et al. 2012), we predicted that the activity in the SN/VTA, which contains dopaminergic neurons also projecting to the hippocampus (Lisman et al. 2011), would be related to that memory benefit. However, fMRI analyses on the basis of subsequent memory performance (using the memory parameter) did not implicate the SN/VTA. Instead, as in previous studies (Guitart-Masip et al. 2011, 2012), activity in the SN/VTA as well as striatal activity were overall higher for a requirement to act (Fig. 3). Thus, while this overall effect of action in the SN/VTA and striatum mirrored the memory benefit of action, the functional relationship between this brain activity pattern and subsequent memory remained indirect.

In addition to a main effect of action, the behavioral memory performance after 24 h showed an interaction pattern of Pavlovian congruence (Guitart-Masip et al. 2014), favoring Go Win (motor activation to obtain rewards) and No-Go Avoid (motor inhibition when faced with punishment), over the incongruent conditions (No-Go Win and Go Avoid). The only region in the basal ganglia that showed a neural interaction effect consistent with Pavlovian congruence was the right dorsal putamen. A cluster in this region predicted memory fate more in the congruent than the incongruent conditions (Fig. 4). Of course, the finding of a neural interaction effect does not mean that this signal is necessary to produce the behavioral results. To our knowledge, there is no direct projection between the dorsal parts of the putamen and the hippocampus (Friedman et al. 2002; Haber and Knutson 2010). Therefore, a memory-related modulation of activity was not predicted for this region. However, it is interesting to note that a decreased volume of the putamen has recently also been observed, albeit not predicted, in preclinical stages of dominantly inherited Alzheimer's disease (Cash et al. 2013), a disorder where memory problems are among the first symptoms. Also, studies in non-human primates show that bilateral lesions to the entorhinal and perirhinal cortices, important input/output structures of the hippocampus, lead to metabolic dysfunction in the basal ganglia including the putamen (Meguro et al. 1999). Taken together, our observations [also see Sadeh et al. (2011)] and the data regarding Alzheimer's disease point to the possibility that functions related to motor control in the putamen also influence the ability to recognize or recollect events in long-term memory. This adds to an emerging understanding of Pavlovian control in the striatum (Guitart-Masip et al. 2014) and a role for putamen to factor in the nature of an anticipated, instrumental action (Kurniawan et al. 2013). The results also expand the knowledge of striatal contributions to memory (Scimeca and Badre 2012) and suggest that there is clinical importance in investigating the interaction between memory and action.

Previous experiments have highlighted a benefit of reward expectation on long-term memory (Wittmann et al. 2005; 2011; Adcock et al. 2006), yet did not fully orthogonalize valence and action (Guitart-Masip et al. 2014). Our behavioral results now show that on trials that were associated with a positive expectation of value (Win conditions), the need to act enhanced memory performance compared with the need to withhold action (Fig. 2). These observations highlight the importance of fully controlling modes of action and types of outcomes to understand the motivational regulation of long-term memory, particularly with regard to the role of the hippocampus. Further research might usefully determine how action and inaction relate to effort and the associated costs of acting. While on the one hand effortful actions may be discounted (Kurniawan et al. 2011), in other circumstances effort can be associated with increased evaluation (Zentall 2010).

Our data highlight a hippocampal role in instrumentally omitted actions (see Supplementary Materials for similar results obtained in previous studies). The neural network pattern of inaction (Fig. 3, also see Supplementary Material) is not consistent with the default network (Buckner et al. 2008), which one may expect if subjects were passively resting during No-Go. Indeed, the hippocampus has been associated with avoidance of potential threat (Bach et al. 2014; Bannerman et al. 2014), the expression of memory-dependent on behavioral inhibition (Bannerman et al. 2012), and inhibitory control of action for reward (Chudasama et al. 2012; Abela et al. 2013; Abela and Chudasama 2014). Another possibility which chimes well with recent accounts of hippocampal–basal ganglia interactions (Shohamy and Turk-Browne 2013) is that the hippocampus can modulate basal ganglia function on the basis of declarative memory (Pennartz et al. 2011). Another possibility is that hippocampally recalled task rules compete with action biases (Izquierdo et al. 2006; Eschenko and Mizumori 2007; Doll et al. 2009; Peyrache et al. 2009). Irrespective of which of these interpretations is true, these findings add to an emerging perspective of the broader impact the hippocampus has on cognition (Shohamy and Turk-Browne 2013) and its potentially antagonistic function to the basal ganglia under certain circumstances (Seger and Cincotta 2006; Lee et al. 2008; Foerde et al. 2013). In the current design, embedding the task in the semantic content of stimuli ensured the same level of stimulus-bound attention for each image. The requirement to perform an action in the Go- but not in the No-Go trials may have induced an additional attentional modulation directed toward the subsequent target detection task. While we cannot fully exclude the possibility that this additional action-related requirement had an effect on memory, we note evidence from levels-of-processing studies indicate that attention per se cannot improve memory if it is not directed toward the semantic content of a stimulus (Craik 2002). Likewise, an action-related attentional effect could not account for the interaction pattern observable in the behavioral and neural data that integrates action, valence, and memory.

The strong memory difference between action for reward and inaction for reward has implications for how we interact with information toward optimizing long-term learning. Indeed, the advantages of “active” over “passive” learning approaches have been of interest to educational psychologists (Michael 2006). While the “active” component in active learning approaches obviously exceeds the experimentally controlled and deliberately simple motor responses required here, our findings provide a neurocognitive framework that can account for the benefits of active learning. Most strikingly, we show that the omission of an action for reward is the least conducive to persistence of memory. Indeed, many educational approaches are likely to be suboptimal for memory consolidation because they are based on passive positive reinforcement.

In conclusion, we show a long-lasting modulation of recognition memory and recollection by the influence of anticipated action and the Pavlovian congruence between action and reward. This modulation is associated with complementary contributions of the basal ganglia and the hippocampus to action anticipation and the omission of action. How the 2 regions functionally interact to enable the observed modulation of memory consolidation remains to be established because previously reported functional interactions between the hippocampus and the SN/VTA do not fully account for the behavioral effect while the putamen, a region without known anatomical connectivity to the hippocampus, does. Our findings therefore indicate a novel type of functional division between the hippocampus and basal ganglia in the motivational regulation of long-term memory consolidation, which favors remembering events that are worth acting for.

## Supplementary Material

Supplementary material can be found at: http://www.cercor.oxfordjournals.org/.

## Funding

This work was supported by an UCL Grand Challenge Studentship in Biomedicine to R.K., the Wellcome Trust (Senior Investigator Award 098362/Z/12/Z to R.J.D.), and DFG grant SFB 779 (A07) supporting E.D. The Wellcome Trust Centre for Neuroimaging is supported by core funding from the Wellcome Trust 091593/Z/10/Z. Funding to pay the Open Access publication charges for this article was provided by the The Wellcome Trust.

## Supplementary Material

Supplementary Data

## References

[BHV216C1] Abela AR, Chudasama Y 2014 Noradrenergic alpha2A-receptor stimulation in the ventral hippocampus reduces impulsive decision-making. Psychopharmacology (Berl). 231 (3):521–531. 24062084 10.1007/s00213-013-3262-y

[BHV216C2] Abela AR, Dougherty SD, Fagen ED, Hill CJ, Chudasama Y 2013 Inhibitory control deficits in rats with ventral hippocampal lesions. Cereb Cortex. 23 (6):1396–1409. 22615141 10.1093/cercor/bhs121

[BHV216C3] Adcock RA, Thangavel A, Whitfield-Gabrieli S, Knutson B, Gabrieli JD 2006 Reward-motivated learning: mesolimbic activation precedes memory formation. Neuron. 50 (3):507–517. 16675403 10.1016/j.neuron.2006.03.036

[BHV216C4] Bach DR, Guitart-Masip M, Packard PA, Miró J, Falip M, Fuentemilla L, Dolan RJ 2014 Human hippocampus arbitrates approach-avoidance conflict. Cur Biol. 24:541–547. 10.1016/j.cub.2014.01.046 PMC3969259 24560572

[BHV216C5] Bannerman DM, Bus T, Taylor A, Sanderson DJ, Schwarz I, Jensen V, Hvalby O, Rawlins JN, Seeburg PH, Sprengel R 2012 Dissecting spatial knowledge from spatial choice by hippocampal NMDA receptor deletion. Nat Neurosci. 15 (8):1153–1159. 22797694 10.1038/nn.3166 PMC3442238

[BHV216C6] Bannerman DM, Sprengel R, Sanderson DJ, McHugh SB, Rawlins JN, Monyer H, Seeburg PH 2014 Hippocampal synaptic plasticity, spatial memory and anxiety. Nat Rev Neurosci. 15 (3):181–192. 24552786 10.1038/nrn3677

[BHV216C7] Brett M, Anton J, Valabregue R, Poline J 2002 Region of interest analysis using an SPM toolbox. Neuroimage. 16 (Suppl 1):1141.

[BHV216C8] Bromberg-Martin ES, Matsumoto M, Hikosaka O 2010 Dopamine in motivational control: rewarding, aversive, and alerting. Neuron. 68 (5):815–834. 21144997 10.1016/j.neuron.2010.11.022 PMC3032992

[BHV216C9] Buckner RL, Andrews-Hanna JR, Schacter DL 2008 The brain's default network: anatomy, function, and relevance to disease. Ann N Y Acad Sci. 1124:1–38. 18400922 10.1196/annals.1440.011

[BHV216C10] Callan DE, Schweighofer N 2008 Positive and negative modulation of word learning by reward anticipation. Hum Brain Mapp. 29 (2):237–249. 17390317 10.1002/hbm.20383 PMC6870695

[BHV216C11] Cash DM, Ridgway GR, Liang Y, Ryan NS, Kinnunen KM, Yeatman T, Malone IB, Benzinger TL, Jack CR Jr, Thompson PM et al 2013 The pattern of atrophy in familial Alzheimer disease: volumetric MRI results from the DIAN study. Neurology. 81 (16):1425–1433. 24049139 10.1212/WNL.0b013e3182a841c6 PMC3806583

[BHV216C12] Chowdhury R, Guitart-Masip M, Bunzeck N, Dolan RJ, Duzel E 2012 Dopamine modulates episodic memory persistence in old age. J Neurosci. 32 (41):14193–14204. 23055489 10.1523/JNEUROSCI.1278-12.2012 PMC3734374

[BHV216C13] Chudasama Y, Doobay VM, Liu Y 2012 Hippocampal-prefrontal cortical circuit mediates inhibitory response control in the rat. J Neurosci. 32 (32):10915–10924. 22875926 10.1523/JNEUROSCI.1463-12.2012 PMC6621026

[BHV216C14] Craik F 2002 Levels of processing: past, present… and future? Memory. 10 (5–6):305–318. 12396643 10.1080/09658210244000135

[BHV216C15] Crockett MJ, Clark L, Robbins TW 2009 Reconciling the role of serotonin in behavioral inhibition and aversion: acute tryptophan depletion abolishes punishment-induced inhibition in humans. J Neurosci. 29 (38):11993–11999. 19776285 10.1523/JNEUROSCI.2513-09.2009 PMC2775933

[BHV216C16] Dayan P, Niv Y, Seymour B, Daw ND 2006 The misbehavior of value and the discipline of the will. Neural Netw. 19 (8):1153–1160. 16938432 10.1016/j.neunet.2006.03.002

[BHV216C17] Doll BB, Jacobs WJ, Sanfey AG, Frank MJ 2009 Instructional control of reinforcement learning: a behavioral and neurocomputational investigation. Brain Res. 1299:74–94. 19595993 10.1016/j.brainres.2009.07.007 PMC3050481

[BHV216C18] Duzel E, Bunzeck N, Guitart-Masip M, Wittmann B, Schott BH, Tobler PN 2009 Functional imaging of the human dopaminergic midbrain. Trends Neurosci. 32 (6):321–328. 19446348 10.1016/j.tins.2009.02.005

[BHV216C19] Eichenbaum H, Yonelinas AP, Ranganath C 2007 The medial temporal lobe and recognition memory. Annu Rev Neurosci. 30:123–152. 17417939 10.1146/annurev.neuro.30.051606.094328 PMC2064941

[BHV216C20] Eschenko O, Mizumori SJY 2007 Memory influences on hippocampal and striatal neural codes: effects of a shift between task rules. Neurobiol Learn Mem. 87 (4):495–509. 17240173 10.1016/j.nlm.2006.09.008 PMC1940837

[BHV216C21] Foerde K, Braun EK, Shohamy D 2013 A trade-off between feedback-based learning and episodic memory for feedback events: evidence from Parkinson's disease. Neurodegener Dis. 11 (2):93–101. 23036965 10.1159/000342000

[BHV216C22] Friedman DP, Aggleton JP, Saunders RC 2002 Comparison of hippocampal, amygdala, and perirhinal projections to the nucleus accumbens: combined anterograde and retrograde tracing study in the Macaque brain. J Comp Neurol. 450 (4):345–365. 12209848 10.1002/cne.10336

[BHV216C23] Guitart-Masip M, Chowdhury R, Sharot T, Dayan P, Duzel E, Dolan RJ 2012 Action controls dopaminergic enhancement of reward representations. Proc Natl Acad Sci USA. 109 (19):7511–7516. 22529363 10.1073/pnas.1202229109 PMC3358848

[BHV216C24] Guitart-Masip M, Duzel E, Dolan R, Dayan P 2014 Action versus valence in decision making. Trends Cogn Sci. 18:194–202. 24581556 10.1016/j.tics.2014.01.003 PMC3989998

[BHV216C25] Guitart-Masip M, Fuentemilla L, Bach DR, Huys QJ, Dayan P, Dolan RJ, Duzel E 2011 Action dominates valence in anticipatory representations in the human striatum and dopaminergic midbrain. J Neurosci. 31 (21):7867–7875. 21613500 10.1523/JNEUROSCI.6376-10.2011 PMC3109549

[BHV216C26] Haber SN, Knutson B 2010 The reward circuit: linking primate anatomy and human imaging. Neuropsychopharmacology. 35 (1):4–26. 19812543 10.1038/npp.2009.129 PMC3055449

[BHV216C27] Hansen N, Manahan-Vaughan D 2014 Dopamine D1/D5 receptors mediate informational saliency that promotes persistent hippocampal long-term plasticity. Cereb Cortex. 24 4:845–858. 23183712 10.1093/cercor/bhs362 PMC3948488

[BHV216C28] Hutton C, Josephs O, Stadler J, Featherstone E, Reid A, Speck O, Bernarding J, Weiskopf N 2011 The impact of physiological noise correction on fMRI at 7T. Neuroimage. 57 (1):101–112. 21515386 10.1016/j.neuroimage.2011.04.018 PMC3115139

[BHV216C29] Izquierdo I, Bevilaqua LM, Rossato J, Bonini J, Silva WD, Medina J, Cammarota M 2006 The connection between the hippocampal and the striatal memory systems of the brain: a review of recent findings. Neurotox Res. 10 (2):113–121. 17062373 10.1007/BF03033240

[BHV216C30] Kurniawan IT, Guitart-Masip M, Dayan P, Dolan RJ 2013 Effort and valuation in the brain: the effects of anticipation and execution. J Neurosci. 33 (14):6160–6169. 23554497 10.1523/JNEUROSCI.4777-12.2013 PMC3639311

[BHV216C31] Kurniawan IT, Guitart-Masip M, Dolan RJ 2011 Dopamine and effort-based decision making. Front Neurosci. 5:81 doi:10.3389/fnins.2011.00081. 21734862 10.3389/fnins.2011.00081 PMC3122071

[BHV216C32] Lancaster JL, Woldorff MG, Parsons LM, Liotti M, Freitas CS, Rainey L, Kochunov PV, Nickerson D, Mikiten SA, Fox PT 2000 Automated Talairach atlas labels for functional brain mapping. Hum Brain Mapp. 10 (3):120–131. 10912591 10.1002/1097-0193(200007)10:3<120::AID-HBM30>3.0.CO;2-8 PMC6871915

[BHV216C33] Lee AS, Duman RS, Pittenger C 2008 A double dissociation revealing bidirectional competition between striatum and hippocampus during learning. Proc Natl Acad Sci USA. 105 (44):17163–17168. 18955704 10.1073/pnas.0807749105 PMC2579395

[BHV216C34] Levita L, Hoskin R, Champi S 2012 Avoidance of harm and anxiety: a role for the nucleus accumbens. Neuroimage. 62 (1):189–198. 22569544 10.1016/j.neuroimage.2012.04.059

[BHV216C35] Lisman J, Grace AA, Duzel E 2011 A neoHebbian framework for episodic memory; role of dopamine-dependent late LTP. Trends Neurosci. 34 (10):536–547. 21851992 10.1016/j.tins.2011.07.006 PMC3183413

[BHV216C36] Lisman JE, Grace AA 2005 The hippocampal-VTA loop: controlling the entry of information into long-term memory. Neuron. 46 (5):703–713. 15924857 10.1016/j.neuron.2005.05.002

[BHV216C37] Lutti A, Hutton C, Finsterbusch J, Helms G, Weiskopf N 2010 Optimization and validation of methods for mapping of the radiofrequency transmit field at 3T. Magn Reson Med. 64 (1):229–238. 20572153 10.1002/mrm.22421 PMC3077518

[BHV216C38] Maldjian JA, Laurienti PJ, Kraft RA, Burdette JH 2003 An automated method for neuroanatomic and cytoarchitectonic atlas-based interrogation of fMRI data sets. Neuroimage. 19 (3):1233–1239. 12880848 10.1016/s1053-8119(03)00169-1

[BHV216C39] Meguro K, Blaizot X, Kondoh Y, Le Mestric C, Baron JC, Chavoix C 1999 Neocortical and hippocampal glucose hypometabolism following neurotoxic lesions of the entorhinal and perirhinal cortices in the non-human primate as shown by PET. Implications for Alzheimer's disease. Brain 122 (Pt 8):1519–1531. 10430835 10.1093/brain/122.8.1519

[BHV216C40] Michael J 2006 Where's the evidence that active learning works? Adv Physiol Ed. 30 (4):159–167. 10.1152/advan.00053.2006 17108243

[BHV216C41] Morris RG 2006 Elements of a neurobiological theory of hippocampal function: the role of synaptic plasticity, synaptic tagging and schemas. Eur J Neurosci. 23 (11):2829–2846. 16819972 10.1111/j.1460-9568.2006.04888.x

[BHV216C42] O'Carroll CM, Martin SJ, Sandin J, Frenguelli B, Morris RG 2006 Dopaminergic modulation of the persistence of one-trial hippocampus-dependent memory. Learn Mem. 13 (6):760–769. 17142305 10.1101/lm.321006 PMC1783630

[BHV216C43] Pennartz CM, Ito R, Verschure PF, Battaglia FP, Robbins TW 2011 The hippocampal-striatal axis in learning, prediction and goal-directed behavior. Trends Neurosci. 34 (10):548–559. 21889806 10.1016/j.tins.2011.08.001

[BHV216C44] Peyrache A, Khamassi M, Benchenane K, Wiener SI, Battaglia FP 2009 Replay of rule-learning related neural patterns in the prefrontal cortex during sleep. Nat Neurosci. 12 (7):919–926. 19483687 10.1038/nn.2337

[BHV216C45] Ranganath C 2010 A unified framework for the functional organization of the medial temporal lobes and the phenomenology of episodic memory. Hippocampus. 20 (11):1263–1290. 20928833 10.1002/hipo.20852

[BHV216C46] Sadeh T, Shohamy D, Levy DR, Reggev N, Maril A 2011 Cooperation between the hippocampus and the striatum during episodic encoding. J Cogn Neurosci. 23 (7):1597–1608. 20666593 10.1162/jocn.2010.21549

[BHV216C47] Schonberg T, Bakkour A, Hover AM, Mumford JA, Nagar L, Perez J, Poldrack RA 2014 Changing value through cued approach: an automatic mechanism of behavior change. Nat Neurosci. 17 (4):625–630. 24609465 10.1038/nn.3673 PMC4041518

[BHV216C48] Scimeca JM, Badre D 2012 Striatal contributions to declarative memory retrieval. Neuron. 75 (3):380–392. 22884322 10.1016/j.neuron.2012.07.014 PMC3432931

[BHV216C49] Seger CA, Cincotta CM 2006 Dynamics of frontal, striatal, and hippocampal systems during rule learning. Cereb Cortex. 16 (11):1546–1555. 16373455 10.1093/cercor/bhj092

[BHV216C50] Shohamy D, Adcock RA 2010 Dopamine and adaptive memory. Trends Cogn Sci. 14 (10):464–472. 20829095 10.1016/j.tics.2010.08.002

[BHV216C51] Shohamy D, Turk-Browne NB 2013 Mechanisms for widespread hippocampal involvement in cognition. J Exp Psychol Gen. 142 (4):1159–1170. 24246058 10.1037/a0034461 PMC4065494

[BHV216C52] Squire LR, Wixted JT 2011 The cognitive neuroscience of human memory since H.M. Annu Rev Neurosci. 34:259–288. 21456960 10.1146/annurev-neuro-061010-113720 PMC3192650

[BHV216C53] Tricomi EM, Delgado MR, Fiez JA 2004 Modulation of caudate activity by action contingency. Neuron. 41 (2):281–292. 14741108 10.1016/s0896-6273(03)00848-1

[BHV216C54] Vargha-Khadem F, Gadian DG, Watkins KE, Connelly A, Van Paesschen W, Mishkin M 1997 Differential effects of early hippocampal pathology on episodic and semantic memory. Science. 277 (5324):376–380. 9219696 10.1126/science.277.5324.376

[BHV216C55] Weiskopf N, Suckling J, Williams G, Correia MM, Inkster B, Tait R, Ooi C, Bullmore ET, Lutti A 2013 Quantitative multi-parameter mapping of R1, PD(*), MT, and R2(*) at 3T: a multi-center validation. Front Neurosci. 7:95. 23772204 10.3389/fnins.2013.00095 PMC3677134

[BHV216C56] Wittmann BC, Dolan RJ, Duzel E 2011 Behavioral specifications of reward-associated long-term memory enhancement in humans. Learn Mem. 18 (5):296–300. 21502336 10.1101/lm.1996811 PMC3465832

[BHV216C57] Wittmann BC, Schott BH, Guderian S, Frey JU, Heinze HJ, Duzel E 2005 Reward-related fMRI activation of dopaminergic midbrain is associated with enhanced hippocampus-dependent long-term memory formation. Neuron. 45 (3):459–467. 15694331 10.1016/j.neuron.2005.01.010

[BHV216C58] Wolosin SM, Zeithamova D, Preston AR 2012 Reward modulation of hippocampal subfield activation during successful associative encoding and retrieval. J Cogn Neurosci. 24 (7):1532–1547. 22524296 10.1162/jocn_a_00237 PMC3393089

[BHV216C59] Zentall TR 2010 Justification of effort by humans and pigeons cognitive dissonance or contrast? Curr Dir Psychol. 19 (5):296–300.

[BHV216C60] Zink CF, Pagnoni G, Martin-Skurski ME, Chappelow JC, Berns GS 2004 Human striatal responses to monetary reward depend on saliency. Neuron. 42 (3):509–517. 15134646 10.1016/s0896-6273(04)00183-7

