# Supplementary material for: Basal Ganglia Activity Mirrors a Benefit of Action and Reward on Long-Lasting Event Memory
Source: Cereb Cortex. 2015 Sep 28;25(12):4908–17. doi: 10.1093/cercor/bhv216 (PMC4635928; doi:10.1093/cercor/bhv216)
Supplement: Supplementary Data [file supp_25_12_4908__index.html]

Basal Ganglia Activity Mirrors a Benefit of Action and Reward on Long-Lasting Event Memory — Supplementary Data 

# Basal Ganglia Activity Mirrors a Benefit of Action and Reward on Long-Lasting Event Memory

## Supplementary Data

Supplementary Data

- Supplementary Data - Docx file
