# Supplementary material for: Basal Ganglia Activity Mirrors a Benefit of Action and Reward on Long-Lasting Event Memory
Source: Cereb Cortex. 2015 Sep 28;25(12):4908–17. doi: 10.1093/cercor/bhv216 (PMC4635928; doi:10.1093/cercor/bhv216)
Supplement: Supplementary Data [file supp_bhv216_bhv216supp.docx]

**Supplementary Materials**

**Additional behavioral memory measures**

In order to analyze recognition memory, the percentage of falsely recognized images of a condition (number of recognition responses to unseen images of a condition divided by the total number of unseen images in this condition) was subtracted from the percentage of correctly recognized images of this condition (number of recognized images for which the action requirement was solved correctly in a condition divided by the number of all seen images in this condition for which the action requirement was solved correctly) to form the corrected hit rate per condition (as done previously by e.g. Wittmann et al., 2011). The corrected hit rate was analyzed with a 2x2 repeated-measures ANOVA with action (Go/No-Go) and valence (Win/Avoid) as factors (the same procedure was used for the ‘Remember’ responses). The corrected hitrate shows an insignificant trend for the main effect of enhancement by action (F(1,28)=4.04, p=.054) and a significant interaction favoring the congruent conditions (F(1,28)=5.16, p=.031) with no effect of valence (F(1,28)=1.63, p>.2). These results show that all employed memory measures show a highly similar pattern.

**Additional activations of memory, action and inaction**

**Effect of inaction in previous studies**

Supplementary Figure 1. displays the contrast of No Go against Go showed activation in the hippocampus in previous studies, using a similar design in which the cue images were fractal cues (Guitart-Masip et al., 2012; Guitart-Masip et al., 2011). While this specific contrast was not reported initially, these activations are consistent with the pattern found in the current study.


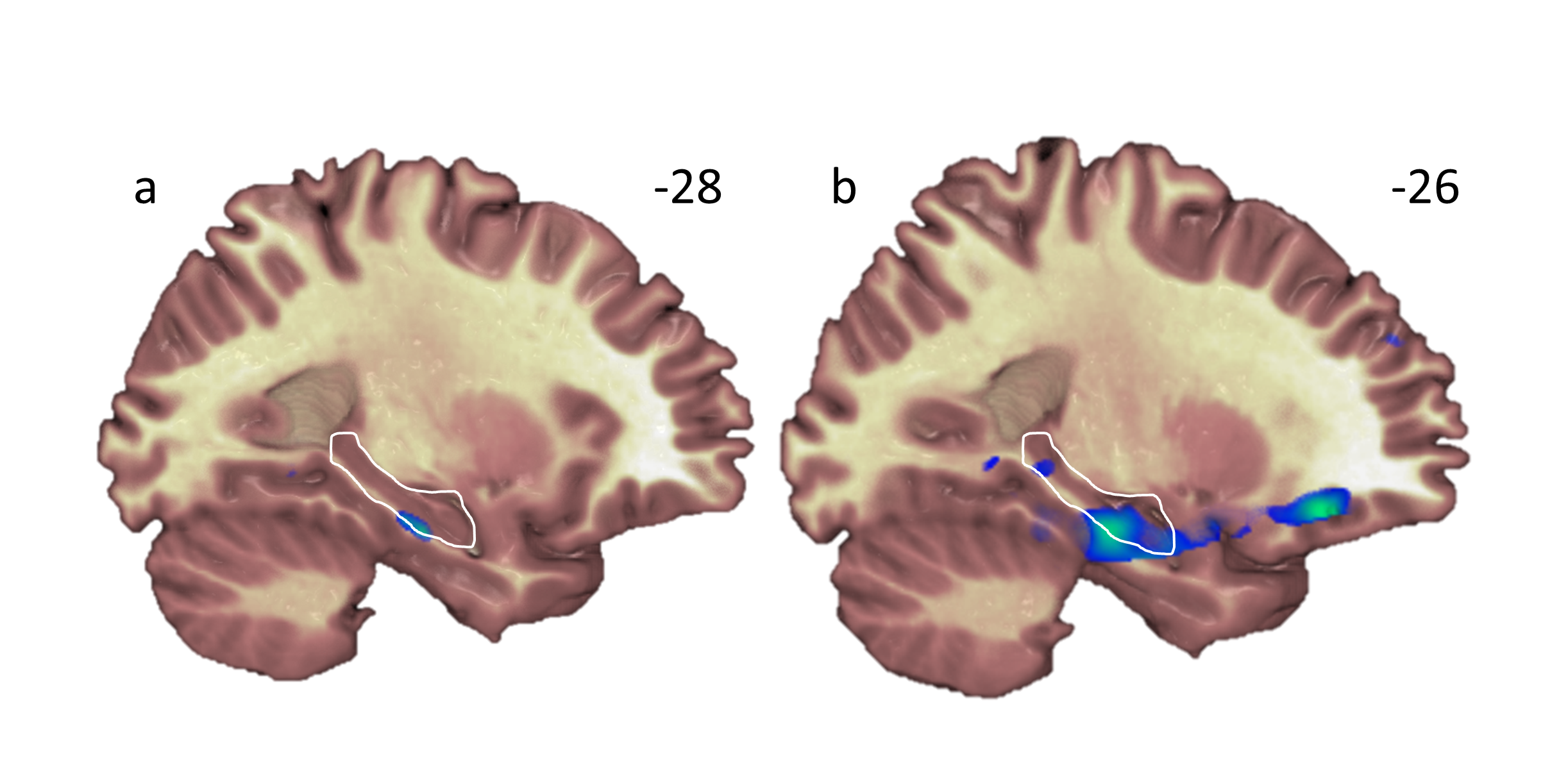


Supplementary Figure 1. The main effect of inaction is displayed at p<.001 uncorrected. The left hippocampus is outlined. This contrast was obtained in previous studies (a: Guitart-Masip et al. (2011), b: Guitart-Masip et al. (2012) but was not reported.

**Parameter estimates in the hippocampus**

As the hippocampus revealed no differences for the memory parameter across the four conditions, we extracted the beta parameters for all conditions to investigate the full picture of the activation pattern. As shown in Supplementary Figure 2, extracting the betas for both the cue activity and memory parameter revealed a significant effect of inaction on the cue activity (F(1,28)=8.3, p=.008, with the No Go Avoid condition showing more activation than the Go Win (t(28)=2.35, p=.026) and Go Avoid condition (t(28)=3.57, p=.001), see also Supplementary Figure 2b). The memory parameter (see Supplementary Figure 2c) in the No Go Avoid condition was larger than in the Go Win (t(28)=2.73, p=.011), Go Avoid (t(28)=2.38, p=.025) and No Go Win (t(28)=2.6, p=.015) condition (no significant main effect, F(1,28)=2.3, p=.14; or interaction, F(1,28)=2.09, p=.16). This shows a dissociation from the behavioral pattern in which the Go Win condition is significantly stronger than the No Go Avoid condition. The cluster shows a positive memory parameter for every condition except the No Go Win condition (Go Win: t(28)=2.59, p=.015, Go Avoid: t(28)=2.7, p=.012, No Go Win: t(28)=1.66, p=.11, No Go Avoid: t(28)=6.01, p<.001)

Supplementary Figure 2. a. Activity associated with the memory parameter is displayed at p<.001 uncorrected. The betas of the left hippocampus cluster for cue activity (b) and the memory parameter (c) were extracted. The cue activity shows the main effect of action observed in large parts of the hippocampus. The memory parameter in the No Go Avoid condition is significantly larger than in every other condition. Error bars represent standard error of the mean. Significant differences (p<.05) are marked by an asterisk


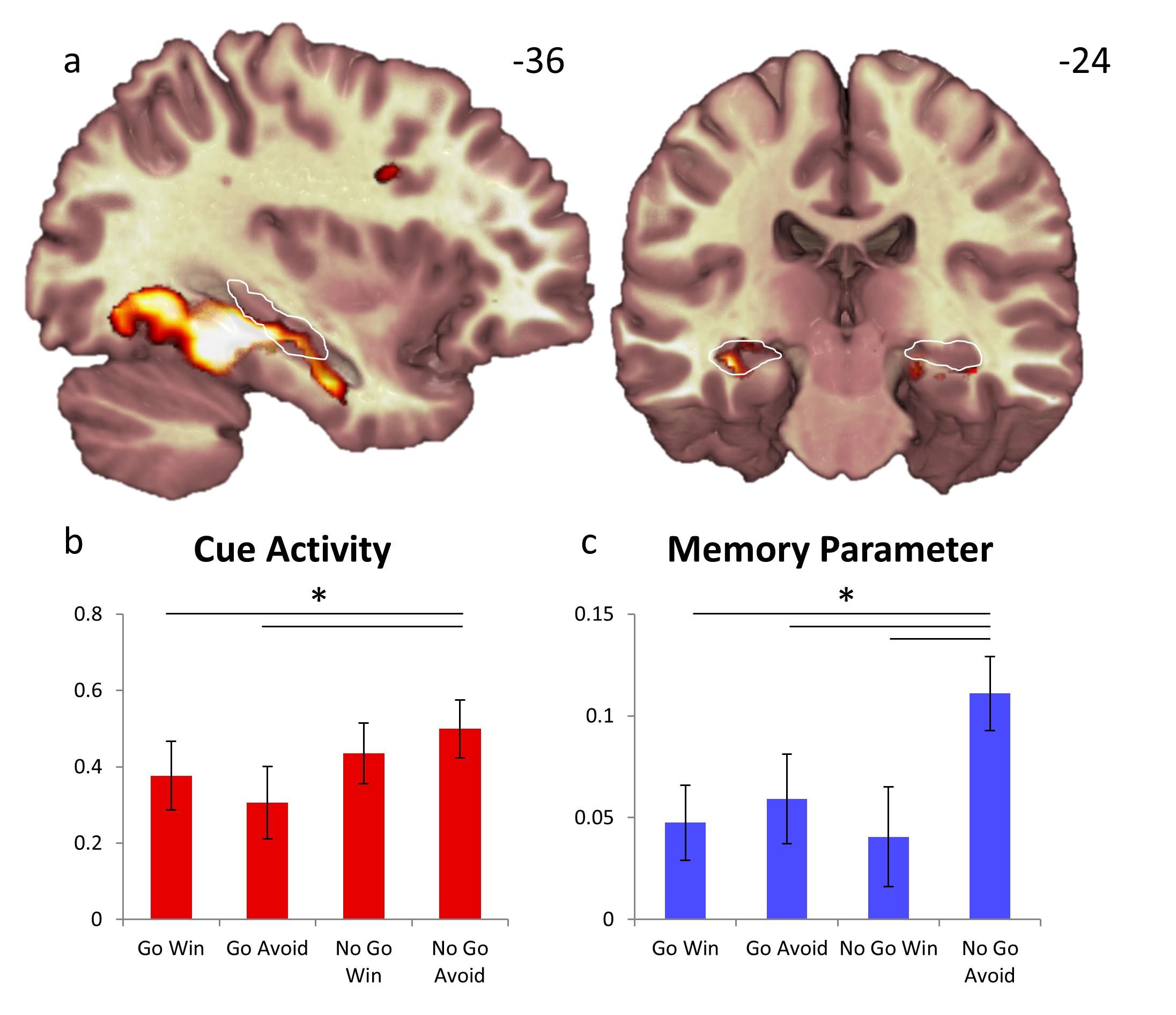


**Interaction of action and valence in the SN/VTA**

Surprisingly, the analysis presented in the main text indicated that the putamen was the only region that showed an interaction between action and valence on the memory parameter. A priori, we would have expected that the SN/VTA would show such an effect given its previously established role in the motivational regulation of memory consolidation (Adcock et al. 2006; Wittmann et al. 2005). We therefore conducted a more sensitive follow-up analysis specifically for this region. In this analysis, we first investigated whether the SN/VTA showed action and valence effects in the cue activity (irrespective of subsequent memory). A significant cluster in the dorsolateral SN/VTA showed an interaction between action and valence (Supplementary Figure 3. small-volume corrected (SVC) for bilateral anatomical SN/VTA, peaking at 16 -22 -6, p=.019, z=3.64, k=5). As observed in the more medial portions of the SN/VTA this cluster also showed a main effect of action (F(1,28)=28.1, p<.001). Comparing the extracted parameter estimates for this cluster in the dorsolateral SN/VTA with the more medial SN/VTA cluster selected for anticipation of action (as displayed in Figure 3c in the main text) showed a significantly (F(1,28)=13.73, p<.001) larger interaction of action and valence in the dorsolateral SN/VTA. We then refocused on the memory parameter in this region and found that, remarkably, the dorsolateral SN/VTA cluster showed a significant memory parameter the Go Win condition (t(28)=2.14, p=.041). This is consistent with previous findings on SN/VTA involvement in reward related memory enhancement (Adcock et al. 2006; Wittmann et al. 2005). The pattern of the memory parameter differed across the two SN/VTA subregions (interaction of action, valence and region: F(1,28)=4.16, p=.05; difference for Go Win: t(28)=3.65, p=.001), however not across conditions within the dorsolateral SN/VTA (main effects and interaction p>.1). These results thus show that while the SN/VTA has a specific relationship to memory consolidation in the Go/Win condition, its activity does not fully account for the combined memory benefit of action and Pavlovian congruence.

Supplementary Figure 3. a. The main effect of action is displayed in red and the interaction of action and valence is displayed in green on an average of all normalized magnetization transfer images (both at p<.001 uncorrected). b. The extracted betas of the cue activity in the dorsolateral cluster of the SN/VTA (green) show the interaction of action and valence for which it was selected and a main effect of action (p<.001). c. The memory parameter estimate for the Go Win condition is significantly larger than zero. The activation patterns in the dorsolateral SN/VTA differ significantly from the more medial subregion.


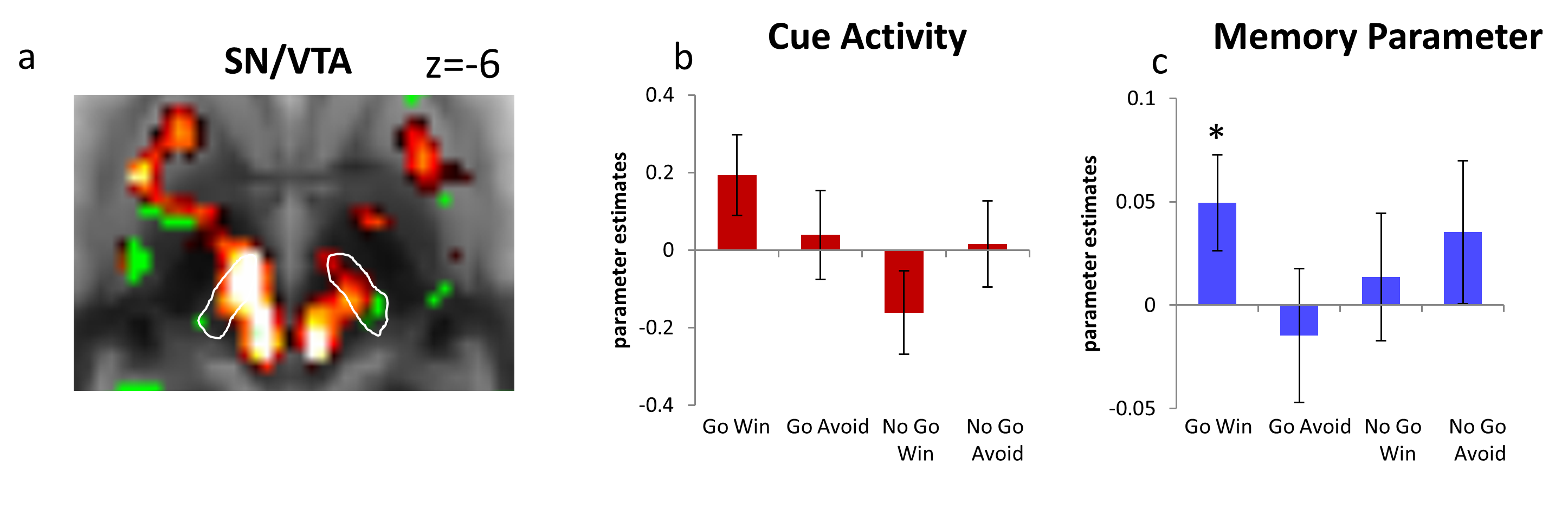


**Functional coupling of SN/VTA and hippocampus**

A psychophysiological interaction (PPI) analysis was performed in order to investigate the functional coupling of the SN/VTA and hippocampus. The seed was the dorsolateral SN/VTA cluster selected for an interaction effect of the cue activity. The model consisted of: 1. The activation time course of the volume of interest (dorsolateral SN/VTA). 2. A regressor representing the psychological variable of interest (contrasting cue activity of the four conditions and the related memory parameters, containing the activity at the time of encoding, over all other modeled conditions, representing the baseline). 3. A regressor representing the cross product of the previous two (the psychophysiological interaction term). The first 2 regressors were added as covariates to the model whilst the last regressor was the regressor of interest. As functional coupling with the hippocampus was of specific interest the PPI contrast was assessed by small volume correction for the bilateral anatomical hippocampus after thresholding at p<.001 uncorrected.

We tested whether functional connectivity between the SN/VTA and the hippocampus may be more directly related to the memory profile, i.e. the subsequent memory performance for each cue (memory parameter). This PPI did not reveal functional connectivity to the hippocampus or other areas implicated in memory. To ensure this finding was not due to an overall lack of sensitivity in our experiment and fMRI protocol, we performed a second PPI in which the psychological variable consistent of all encoding events (this included the overall activity at cue image onsets and the associated memory parameters) weighted against all other modeled events (onsets of circles prompting action and onset of outcomes). This tested whether the functional coupling between the two regions was increased by the activity at the time of encoding. The PPI contrast revealed a significant cluster in the left hippocampus (SVC for bilateral anatomical hippocampus, peaking at -24 -26 -8, p=.048, z=3.89, k=6). Extracted beta estimates of this region showed an interaction effect of in the memory parameter (F(1,28)=5.8, p=.023; congruent conditions have stronger memory parameters than incongruent conditions, driven by the No Go Avoid condition (No Go Avoid: t(28)=3.82, p<.001), but no main effect of action (F(1,28)=.24, p=.63). We also found no difference in connectivity strength between conditions.

Our results show that more targeted analyses of SN/VTA activity revealed a neural interaction pattern in its dorsolateral portion (Supplementary Figure 3) corresponding to the observed behavior. A closer analysis of subsequent memory effects showed that the dorsolateral SN/VTA predicted memory fate of the seen cues in the Go Win condition (Supplementary Figure 3c) but not the other conditions. Interestingly, recent findings in monkeys showed that dorsolateral SN neurons were activated by cognitive demands of a cue image, chiming well with the cognitive demands that were required to translate our unique picture stimuli into action/valence contingencies (Matsumoto and Takada 2013). Observations of functional interaction between the hippocampus and SN/VTA in both humans (Adcock et al. 2006; Kahn and Shohamy 2013; Krebs et al. 2011; Shohamy and Wagner 2008) and rats (Helbing et al. 2013) motivated us to also assess trial-by-trial connectivity between the SN/VTA and hippocampus. We found that connectivity between the dorsolateral SN/VTA and the hippocampus was indeed increased during the time of encoding. However we did not find this connectivity to be predictive of memory or linked to the modulation by action/valence. In combination, our results suggest that a robust enhancement of memory by action cannot be accounted for by established mechanisms like differential activation of hippocampus, SN/VTA or their connectivity (Adcock et al. 2006; Krebs et al. 2011; Shohamy and Wagner 2008; Wittmann et al. 2007)

**References**

Adcock RA, Thangavel A, Whitfield-Gabrieli S, Knutson B, Gabrieli JD. 2006. Reward-motivated learning: mesolimbic activation precedes memory formation. Neuron 50(3):507-17.

Helbing C, Werner G, Angenstein F. 2013. Variations in the temporal pattern of perforant pathway stimulation control the activity in the mesolimbic pathway. Neuroimage 64:43-60.

Kahn I, Shohamy D. 2013. Intrinsic connectivity between the hippocampus, nucleus accumbens, and ventral tegmental area in humans. Hippocampus 23(3):187-192.

Krebs RM, Heipertz D, Schuetze H, Duzel E. 2011. Novelty increases the mesolimbic functional connectivity of the substantia nigra/ventral tegmental area (SN/VTA) during reward anticipation: Evidence from high-resolution fMRI. Neuroimage 58(2):647-55.

Matsumoto M, Takada M. 2013. Distinct representations of cognitive and motivational signals in midbrain dopamine neurons. Neuron 79(5):1011-24.

Shohamy D, Wagner AD. 2008. Integrating memories in the human brain: hippocampal-midbrain encoding of overlapping events. Neuron 60(2):378-89.

Wittmann BC, Bunzeck N, Dolan RJ, Duzel E. 2007. Anticipation of novelty recruits reward system and hippocampus while promoting recollection. Neuroimage 38(1):194-202.

Wittmann BC, Schott BH, Guderian S, Frey JU, Heinze HJ, Duzel E. 2005. Reward-related FMRI activation of dopaminergic midbrain is associated with enhanced hippocampus-dependent long-term memory formation. Neuron 45(3):459-67.
